# Evidence of automatic processing in sequence learning using process-dissociation

**DOI:** 10.2478/v10053-008-0107-z

**Published:** 2012-05-21

**Authors:** Heather M. Mong, David P. McCabe, Benjamin A. Clegg

**Affiliations:** 1 Department of Psychology, Colorado State University, Fort Collins, CO, USA

**Keywords:** implicit learning, sequence learning, process-dissociation, consciousness

## Abstract

This paper proposes a way to apply process-dissociation to sequence learning in addition and extension to the approach used by Destrebecqz and Cleeremans (2001). Participants were trained on two sequences separated from each other by a short break. Following training, participants self-reported their knowledge of the sequences. A recognition test was then performed which required discrimination of two trained sequences, either under the instructions to call any sequence encountered in the experiment “old” (the inclusion condition), or only sequence fragments from one half of the experiment “old” (the exclusion condition). The recognition test elicited automatic and controlled process estimates using the process dissociation procedure, and suggested both processes were involved. Examining the underlying processes supporting performance may provide more information on the fundamental aspects of the implicit and explicit constructs than has been attainable through awareness testing.

## Introduction

The serial reaction time task (SRTT) has become an extremely productive method for studying sequence learning (for reviews, see Abrahamse, Jimenez, Verwey, & Clegg, 2010; Clegg, DiGirolamo, & Keele, 1998). In their original study, Nissen and Bullemer (1987) found that choice reaction time improved to an embedded repeating pattern of locations. Moreover, improvement occurred even without apparent full awareness of the sequence, and such learning was also present in amnesic patients, despite their obvious lack of awareness of the sequence. Although A. Reber (1967) was the first to use the term *implicit learning*, there has been longstanding interest in situations in which learning is apparently unaccompanied by awareness of the material being learned (Ebbinghaus, 1885/1913; Hebb, 1961; Thorndike & Rock, 1934). The distinction between at least two systems is core to a number of theoretical accounts (e.g., Keele, Ivry, Mayr, Hazeltine, & Heuer, 2003; Lewicki, Czyzewska, & Hoffman, 1987; A. Reber, 1989; Willingham & Goedert-Eschmann, 1999).

There have been several reviews of the range of tasks, including the SRTT paradigm, that have been used to examine implicit learning (Berry & Dienes, 1993; Cleeremans & Haynes, 1998; Seger, 1994; Shanks, 2005), and numerous definitions of the term itself have been offered (Dienes & Perner, 1999; Frensch, 1998). This variety is further reflected in an array of methods for assessing the presence or absence of explicit knowledge. Issues raised have included whether explicit knowledge is necessary for learning, what awareness tests should be assessing, and how this could be done (e.g., Cleeremans, 1997; Cleeremans & Haynes, 1998; Destrebecqz & Cleeremans, 2001; Eimer, Goschke, Schlaghecken, & Sturmer, 1996; Frensch & Runger, 2003; Jimenez, Mendez, & Cleeremans, 1996; Perruchet & Amorim, 1992; Perruchet, Bigand, & Benoit-Gonin, 1997; P. Reber, Gitelman, Parrish, & Mesulam, 2003; Reder & Schunn, 1996; Shanks & Johnstone, 1999; Shanks & St. John, 1994; St. John & Shanks, 1997; Zeithamova & Maddox, 2006). In this paper, we propose moving beyond attempts to determine whether participants have any explicit knowledge of a sequence, and rather, shift focus to the nature of the underlying processing.

Tests that attempt to ascertain the level of explicit knowledge have common issues (see Shanks & St. John, 1994), such as potential insensitivity to participants’ explicit knowledge, and face the fundamental challenge that there would be no way to rule out the possibility that some level of awareness exists (Merikle, 1994). A further concern is that explicit knowledge tests are contaminated by implicit knowledge, and vice versa (Neal & Hesketh, 1997).

### Issues in awareness testing

One of the problems with distinguishing between implicit and explicit performance is the very basis on which that distinction can be drawn. There are several ways to assess a participant’s awareness of what has been learned within an implicit learning paradigm. Some awareness testing methods are more readily amenable to specific implicit learning tasks than others, and different tests do have issues that impact how meaningful the results are. One straightforward awareness test is the self-report of awareness of the repeating information presented during the experiment (see Frensch & Runger, 2003; Seth, Dienes, Cleeremans, Overgaard, & Pessoa, 2008; Shanks, 2005). An assumption underlying use of this test is that explicit knowledge can be verbalized, such that a lack of verbalizable knowledge implies implicit learning. One key problem is that a participant may have explicit knowledge, and this information may even be readily available to conscious thought, but the nature of the test may prevent them from describing or conveying this knowledge (Shanks & St. John, 1994).

As discussed by Cheesman and Merikle (1984), self-report tests likely only tap participants’ knowledge of what they know they know (i.e., *metaknowledge*). They argued there are two thresholds important for non-conscious knowledge: a subjective threshold below which participants feel they do not have knowledge, and an objective threshold wherein participants do not feel they have knowledge in addition to not displaying it. Therefore, asking participants to freely verbalize what they know requires the knowledge to be above the subjective threshold.

Dienes and Berry (1997) expanded this idea to implicit learning tasks, including SRTT. By definition, whatever knowledge participants have exceeds the objective threshold, be it implicit or explicit knowledge, and knowledge that participants do not have, is below the objective threshold. Implicit knowledge would be above the objective, but below the subjective threshold. Finally, explicit knowledge would be above both thresholds. They suggested that knowledge acquired through SRTT training is above the objective threshold as participants reliably speed up to the trained sequence. Furthermore, there is evidence that for many participants the acquired knowledge is below the subjective threshold, since conscious knowledge of what was learned is not necessary for the faster reaction times. Self-report tests should tap knowledge available above the subjective threshold, whereas cued tests (i.e., recognition and generation tests) should assess knowledge above the objective threshold, regardless of its relation to the subjective threshold. The distinction between cued and *free report* tests is included in this framework as cued tests should provide a more thorough measure of the knowledge above the objective threshold. Distinguishing whether knowledge is above (i.e., explicit) or below (i.e., implicit) the subjective threshold is naturally much more difficult than determining its relation to the objective threshold. Furthermore, all knowledge above the objective threshold could be employed during cued tests. Consequently, in this framework the results of the cued tests could be thought to provide little distinguishing information on the nature of the underlying knowledge without further manipulation.

One critical point in assessing conscious awareness is that implicit knowledge could be contaminating the explicit knowledge measurements in that the implicit knowledge would inadvertently be used during the explicit measurement (e.g., Shanks & Johnstone, 1999). This is a valid concern that the aforementioned awareness tests are ill-equipped to handle.

### Process-dissociation procedure

If the previously discussed tests necessarily contain contributions from multiple knowledge sources, then a different approach must be adopted. Reingold and Merikle (1988) proposed that, to circumvent needing pure measures of conscious or non-conscious knowledge, the relative sensitivity of two measures could be compared. One measure is a *direct test of knowledge*, in which participants are instructed to make a discrimination (e.g., was an object seen or not). The other measure is an *indirect test of knowledge*, wherein the discrimination is not part of the instructional set. The necessary assumption for this comparison to be valid is that the direct test is more sensitive to conscious knowledge than the indirect test. Therefore, non-conscious knowledge is assumed to be present when discrimination accuracy is higher or reaction times are quicker for the indirect test than the direct test. This concept, originally used in non-conscious perception experiments, was brought into SRTT research by Jimenez et al. (1996), who concluded that not all of the sequence learning could be accounted for by explicit processes. However, while offering some evidence that implicit knowledge appears to be involved, this comparison struggles to satisfactorily address contamination as will be discussed in greater detail below.

Because no memory test, or test of awareness is process-pure, the process-dissociation procedure (PDP; Jacoby, 1991) was developed to estimate the contributions of controlled and automatic processing for implicit memory. There has been recent interest in modifying this procedure to estimate what is happening beyond awareness measures (Destrebecqz & Cleeremans, 2001). Of the instantiations of the procedure within implicit learning (e.g., Buchner, Steffens, Erdfelder, & Rothkegel, 1997), the approach adopted here is most similar to that of Destrebecqz and Cleeremans (2001). This paper uses the distinction of the two-process theory of attention (Schneider & Shiffrin, 1977; Shiffrin & Schneider, 1977), with controlled processes requiring a person’s attentional resources and intent to carry out, and automatic processes occurring without a significant cost to attention and without intent. The direct relation between implicit and explicit learning and the two types of processes is not necessarily clear. However, learning occurring without a person’s awareness can be expected to rely on automatic processes to a greater extent than controlled processes at retrieval.

The rationale behind PDP for decoupling controlled and automatic processing is to create a test condition for which both controlled and automatic processes can lead to a correct response (the inclusion condition), and a test condition in which a failure of controlled processing leads to errors, presumably reflecting automatic processing when controlled processing fails (the exclusion condition). PDP has a long history in implicit memory research (cf. Yonelinas, 2002), and as such has had many refinements to the technique. Therefore, many of the issues have been explored in the literature leading to a stable tool in dissociating controlled from automatic processes.

Similarities between both implicit memory and implicit learning (Buchner, 1994; Buchner & Wippich, 1998) suggest that implicit learning research would benefit from incorporating a dual-process model and approach (Jacoby, Kelley, & Dywan, 1989; Yonelinas, 2002). Thus, using the PDP approach is a natural step in understanding what is happening during implicit learning. Although the PDP approach has been criticized by some researchers (Curran & Hintzman, 1995; Dodson & Johnson, 1996; Graf & Komatsu, 1994), the procedure has proven to be a useful tool across many disciplines within psychology.

The relation of controlled (*C*) and automatic (*A*) processing on the inclusion test is as follows:

*Inclusion* = *C* + (1 - *C*)*A* (1)

By contrast, performance on the exclusion task is accomplished primarily by recollecting items from the study context, which requires controlled processing. In the present study, this involves recollecting which half of training the sequence came from. Thus, errors on the exclusion test reflect *A* in the absence of *C*, which is captured in the following equation:

*Exclusion* = (1 - *C*)*A* (2)

That is, exclusion performance equals the probability that *A* influences responding given that *C* failed to influence responding. In practice, calculating the *C* component of a task is achieved by simply subtracting the false alarm rate for the exclusion condition, which is based on *A* in the absence of *C*, from the hit rate for the inclusion condition, which is based on both *C* and *A*:

*C* = *Inclusion* - *Exclusion* (3)

*A* can then be calculated by dividing false alarms on the exclusion task (automaticity in the absence of control), by the inverse of the controlled process estimate, as shown by solving Equation 2 for *A*:

*A* = Exclusion / (1 - *C*) (4)

Calculating the process estimates avoids the assumption that implicit and explicit processes are used in isolation from each other, and also considers the underlying processing in implicit learning tasks rather than focusing solely on determining the awareness of the participant. The idea that implicit learning might involve automatic processes is not new (cf. e.g., J. Cohen & Poldrack, 2008; Frensch, 1998; Hayes & Broadbent, 1988; Jimenez & Mendez, 1999; Soetens, Melis, & Notebaert, 2004). Furthermore, this is not proposing to solve the debate over how many learning systems are necessary (e.g., Frensch & Runger, 2003; Shanks, 2005; Shanks & St. John, 1994), but instead measuring one part of the whole learning system and its effect on performance.

In the present study, different sequences were employed in the two distinct halves of the training. The inclusion test instructions for a subsequent recognition test asked participants to respond “old” if the presented sequence was from either half of the training phase, and thus, knowledge of either sequence would lead to a correct response; this could result from the influence of either controlled or automatic processing. For the exclusion task instructions, participants responded “old” only if the sequence was from one half of the training phase. Thus, accurate responding in the exclusion condition requires that participants can identify the sequence as having been presented during training, and additionally, requires a temporal discrimination (e.g., Was it from the second half of training?). Consequently, to the extent that controlled processing fails, but automatic processing influences performance, participants will make errors on the exclusion test.

Similar to the memory experiments (Jacoby, 1991; Nairne & Kelley, 2004), the current experiment required participants to discriminate which half of the experiment an item (i.e., sequence) appeared in for the exclusion directions. If participants have control over the sequence knowledge they were trained on, they should be able to successfully make this discrimination. If participants do not have adequate control over this knowledge, then they will be unable to exclude the directed trained sequence as they will be relying on the general familiarity for both sequences in responding. This will lead to a higher automatic processing estimate, and a low or non-existent controlled processing estimate.^1^ For a participant to have a controlled process estimate in this experiment, the participant needs to have access to both the two sequences that were trained, but also the half each sequence was trained. Under the exclusion directions, participants will need the correct temporal cue (i.e., experiment half) associated with the to-be-included or -excluded sequence to make the discrimination. If they are only able to recognize that the sequence had been experienced before, but not at which half, their control over the knowledge for that sequence is incomplete (Shiffrin & Schneider, 1977). In this case, their judgment will be based on the automatic processes supporting familiarity.

There have been concerns about the possibility that participants could have awareness of the learned information, but not have access to when it was learned. One valuable perspective on this issue comes from Yonelinas and Jacoby (1994). This “partial recollection” of the learned information should result in the automatic estimates looking like the controlled. This is because the partial recollections would result in those items being treated as familiar rather than actually recollected, thus bleeding the partially recollected items into the automatic process estimate. It was concluded that this must be infrequent compared to the full recollection rates, and was not a great concern.^2^

In the acquisition phase of implicit learning experiments, participants are usually only exposed to one repeating sequence (Nissen & Bullemer, 1987) or to one set of repeating information (e.g., the repeating visual search arrays of contextual cuing, Chun & Jiang, 1998). Adding a temporal component to testing after non-intentional learning allows for a different criterion of what knowledge is accessible to the participant, as well as a finer grain of analysis of what know-ledge is measurable during testing. Importantly, the introduction of a second sequence within the SRTT paradigm can lead to some temporary short-term interference during its initial acquisition, but it does not impair learning of either sequence (see Stephan, Meier, Orosz, Cattapan-Ludewig, & Kaelin-Lang, 2009).

It has been proposed that the SRTT, and implicit learning research in general, can benefit from shifting to thinking of the underlying pro-cesses for non-intentional learning (e.g., Cleeremans, 1997; Jimenez & Mendez, 2001; Perlman & Tzelgov, 2006, 2009). One conceptualization of an automatic process assumes that the process is capable of occurring without conscious control and without intention (Bargh, 1989, 1992; but see Shanks & Johnstone, 1999). However, all participants will employ both automatic and controlled processes during learning and at test (Buchner & Wippich, 1998). Thus, this study will determine if the standard version of PDP can be implemented within the SRTT paradigm, and based on that investigate the relative influence these two processes have on the task.

### Current experiment

This experiment expanded on the research by Destrebecqz and Cleeremans (2001) in deriving process estimates for trained sequences. Destrebecqz and Cleeremans’ participants were trained on a single sequence, then asked to generate sequence under two instructional forms. The inclusion instructions had participants try to generate the trained sequence, and the exclusion instructions had participants try to generate anything that was not the trained sequence. The logic was that if participants had control over the trained sequence knowledge, they should have minimal intrusions of that information into the exclusion test. However, if this information was out of their control, there would be significant intrusions of the trained sequence in the exclusion gene-ration. By examining the generation proportions under the two instruction sets, Destrebecqz and Cleeremans concluded that participants who were in a condition conducive to gaining explicit awareness and knowledge of the sequence (i.e., a 250 ms pause between responding and the next stimulus) had the ability to control the knowledge gained from sequence training, whereas participants in the condition that did not allow for explicit knowledge to develop (i.e., no pause between responding and the next stimulus) did not have control over the knowledge gained.

The current experiment was conducted to determine if the controlled and automatic processing estimates could be derived (rather than inferring awareness states) from inclusion and exclusion test scores, as well as to find out what experimental set-up would facilitate the PDP calculations. This will allow the discussion of implicit learning to move beyond studying the knowledge gained through training to a deeper level of the processes supporting the knowledge. Examining the control participants have over the knowledge acquired through training can provide new insights into what is changing with learning that may not be possible simply by inferring awareness states. The current experiment can also improve the understanding of what changes with training in sequence learning by allowing the comparison of a participant’s self-reported awareness of learning and their overt control over the acquired knowledge.

Different knowledge tests were used following standard training on two separate sequences. In the current experiment, after training, a self-report knowledge test was administered, in which participants were asked if they noticed any repeating information. Next they were given a recognition PDP test wherein they had to rely on the know-ledge gained during training to be able to appropriately include or exclude the presented sequences. A recognition PDP test was used rather than a generation test for several reasons.^3^ First, with a recognition test, participants will have definitely entered the trained or novel sequence in the cue, whereas for a generation test, it would be possible for participants to never produce the trained sequences. Guaranteeing that participants are re-entering the sequences then allows for a more valid comparison of the participants’ controlled and processes for the trained sequences. That being said, there should not be drastic diffe-rences between ending tests within a modality (Rajaram & Roediger, 1993). Furthermore, both controlled and automatic processes are expected to be used at the ending test as these are posited to support most, if not all, decisions (Jacoby, 1991; Jacoby, Lindsay, & Toth, 1992; Jacoby, Toth, & Yonelinas, 1993).

While there will likely be differences in process estimates between participants, the question remains whether predominant automatic processing can drive enhanced performance with the SRTT. If automatic processes are sufficient for SRTT, participants who did not show evidence of significant controlled processing at test should still have the speeded serial reaction times after acquisition of the trained sequence. Finally, it was expected that overall there would be both controlled and automatic processing estimates; that is, both processes would support the speeded responses on trained sequences.

## Method

### Participants and materials

Forty-six Colorado State University students participated in exchange for partial course credit. All participants had normal or corrected-to-normal vision.

All stimuli were shown and data were collected in E-Prime (Schneider, Eschman, & Zuccolotto, 2002). The SRTT display consisted of a white background with four black square outlines evenly spaced in a row along the center of the display. These four black square outlines essentially served as the place holders for where the target could appear on any trial. Each square was 5 cm along a side, the black outline was 2 mm thick, and the squares all were filled with white. The target was 3.8 cm in diameter, and colored green. It would appear in the center of the appropriate square for each trial.

In total, four ambiguous (A. Cohen, Ivry, & Keele, 1990), second order conditional sequences (Reed & Johnson, 1994) were used in this experiment. All sequences had equivalent response frequencies. The two training sequences were presented to all participants, but in a counterbalanced order. Participants were trained on the sequences shown in the training column of Table 1. Learning of the practiced sequences was assessed through the introduction of new sequences at the eighth block during each training half, known as the *transfer sequences*. These sequences were chosen as they had minimal overlap in similar three and four item response chunks. Importantly, the two training sequences had no three or four overlapping item response chunks.

**Table 1.** Sequences Participants Were Trained on, Transferred to During Training, and Exposed to During the Recognition Test.

Sequence type	
Training	Transfer
1 4 3 1 2 4 2 3 4 1 3 2	2 3 1 3 4 2 1 4 3 2 4 1
1 2 3 1 4 2 1 3 4 3 2 4	4 2 3 4 1 2 4 3 2 1 3 1

*Note*. The leftmost response position is designated by 1, and the rightmost is designated by 4.

### Procedure and measures

Responses were made with the [*v*], [*b*], [*n*], and [*m*] keys, which corresponded to the four display locations from left to right. Participants were instructed to respond as quickly and as accurately as they could. Each trial started when the target appeared in one of the four possible locations and ended when participants made the correct response. After the correct response was made, the target appeared with no delay at the next location. Participants were given four practice trials before starting the experiment.

There were two phases of training blocks, each phase featuring practice with a different 12 item sequence as described above. Each phase had nine blocks of 100 trials each. The first four trials of each block were pseudo-randomly generated and not part of the sequence. Therefore there were 900 trials per half, of those 864 being sequenced trials. There was a mandatory 10 s break between each block, after which participants were free to rest further if they chose. The eighth block switched participants to a non-trained sequence, which was different for the two sets. The ninth block returned participants to the repeated sequence for that section of the experiment. Thus, participants responded to 1,800 stimuli between the two lists, 1,728 of which followed one of the two practiced sequences. In between the two sets, there was a 3 min distracter task.

After participants completed both training sets, their knowledge of the sequences was assessed using two measures. The first was a simple self-report, in which participants were asked, “Did you notice anything repeating? If yes, describe what you noticed. If not, type no to move on.” This was done before the recognition test so the recognition test items did not impact self-reported knowledge. Participants were not explicitly asked to input the trained sequences at this point, they were only asked to describe what patterns they had noticed, if any.

Next, participants completed the recognition PDP task. Participants were informed of the presence of repeating sequences of responses, and that they would now be asked to recognize them. Participants were first told they would be entering part of a sequence (nine items) and then they were asked to respond based on the instructions for that section. The two instruction sets (inclusion and exclusion) were in separate, counterbalanced blocks. Each recognition block started with instructions only for that block. The inclusion instructions told participants to call a sequence fragment “old” if it was from either half of the training period. The exclusion instructions were to call a sequence fragment “old” only if it was from a certain half of the second training set, with half (i.e., first or second) specified by the directions. The half which participants had to exclude was counterbalanced for both which training sequence was viewed first, as well as if the first or second sequence was to be excluded, thus leading to a total of four counterbalance conditions. Unbeknownst to participants, sequences were taken from both sets as well as random sequence fragments. There were 12 trials of each sequence type per instruction set, leading to 96 total trials in the recognition test.

The reaction time for a correct response for all training trials was recorded. The mean reaction time for each block by list was calculated for each participant. Participants’ responses to the recognition test were recorded and likelihood of calling an item “old” was calculated for the different sequence types.

## Results

### Training performance

**Figure 1.**
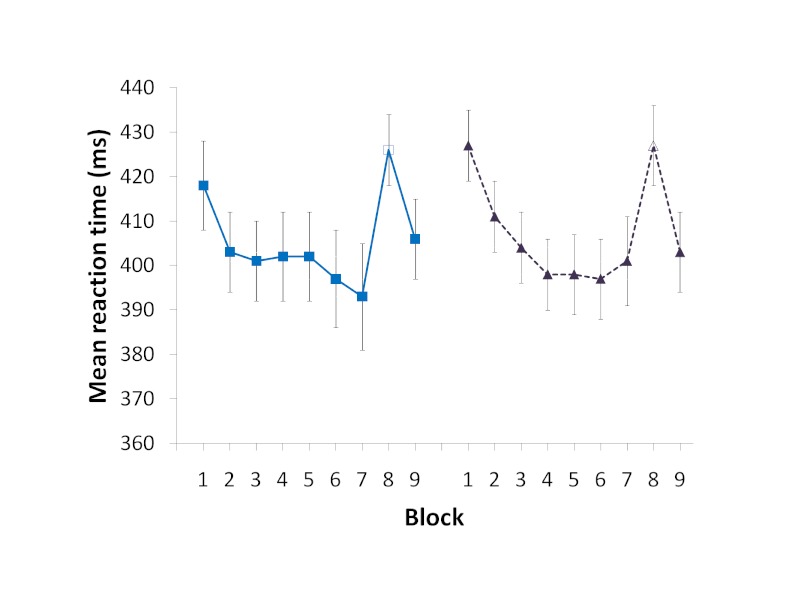
Mean response times in the serial reaction time task (SRTT) by training block and sequence. Closed symbols represent blocks with the trained sequence, and open symbols represent the transfer to a novel sequence. Error bars represent one standard error.

The reaction times from training were submitted to a 2 (training sequence) × 2 (half of training) × 9 (block) repeated-measures MANOVA. The main effect of Block, Wilks’ = .39, *F*(8, 35) = 6.90, *p* < .05, suggests that participants’ performance changed with practice, as shown in Figure 1. There was not a difference by Experimental Half, Wilks’ = .99, *F*(1, 42) = 0.64, *p* > .05. There was also not a diffe-rence between the counterbalance conditions in training performance, *F*(3, 42) = 1.81, *p* > .05, η_p_^2^ = .11.

To test whether participants learned both of the trained sequences, and whether learning was at comparable levels, the mean reaction times for the transfer sequences introduced at the end of training were compared against the mean reaction times of the surrounding trained sequence blocks in a repeated-measures MANOVA. There was a significant difference between the Block Types, Wilks’ = .56, *F*(1, 45) = 35.61, *p* < .05, with participants responding slower on the transfer block. This slower reaction time on the novel sequence indicates that participants were responding faster on the trained sequences because they had learned them, not because they had just gotten more proficient at responding in the task. There was neither no difference between the reaction times for the two experimental halves, Wilks’ = .99, *F*(1, 45) = 0.11, *p* > .05, nor an interaction between the Block Type and Experimental Half (mean reaction time by block type and half in milliseconds: Transfer-Sequence 1 = 422, Trained-Sequence 1 = 395, Transfer-Sequence 2 = 422, Trained-Sequence 2 = 399),Wilks’ = .99, *F*(1, 45) = 0.51, *p* > .05, which is consistent with an equivalent transfer cost across the two halves of the experiment.

### Self-report

Participants were asked at the end of training if they noticed anything repeating during the experiment, and to provide some information on what they noticed. Thirty-one of the 46 participants reported having some level of awareness of the repetition. The remaining 15 non-aware participants showed a significant cost when the trained sequence (mean for the two surrounding trained sequence blocks = 401 ms) was replaced with a novel sequence (*M* = 412 ms), *t*(14) = 2.78, *p* < .05, Cohen’s *d* = 1.05. This finding is congruent with previous suggestions that those participants whose self-reports reflect little awareness of any repeating information in the experiment were nonetheless able to learn the sequences (although as discussed previously, this does not necessarily translate to evidence of purely implicit learning). A first conclusion that can be drawn is that awareness is not necessary for sequence learning, which replicates previous sequence learning findings for the necessity of awareness (e.g., Clegg et al., 1998).

### Recognition test

For the recognition test, participants had been asked to respond to a series of nine locations, as they had during training, but then indicated if this set was “old” or “new” according to the type of instructions. Under inclusion instructions, participants were to call a sequence fragment “old” if they had encountered it during the experiment at any point. The exclusion instructions were to call a sequence fragment “old” only if it was from the second half of the experiment. The likelihood participants called each item type “old” was calculated under both forms of instruction, and is shown in Table 2. In order to calculate PDP estimates, response bias should be roughly equivalent for the inclusion and exclusion tests (Jacoby, 1998)^4^. Response bias was examined by comparing the false alarm rate to the new random sequences, which did not differ for the inclusion and exclusion tests, *t*(45) = 2.0, *p* > .05. There was also not a difference between counterbalance conditions in recognition responses, *F*(3, 42) = 0.16, *p* > .05, η_p_^2^ = .01, and as such will not be discussed further.

**Table 2.** Mean Probability of Responding “Old” to a Sequence Fragment on the Recognition Test by Sequence Type and Instruction Form.

	Sequence type		
	Sequence 1	Sequence 2	Random
Inclusion	.46	.45	.32
Exclusion	.28	.33	.24

*Note*. This is not accuracy, but the proportion of responses for each item type that were “old”.

**Figure 2.**
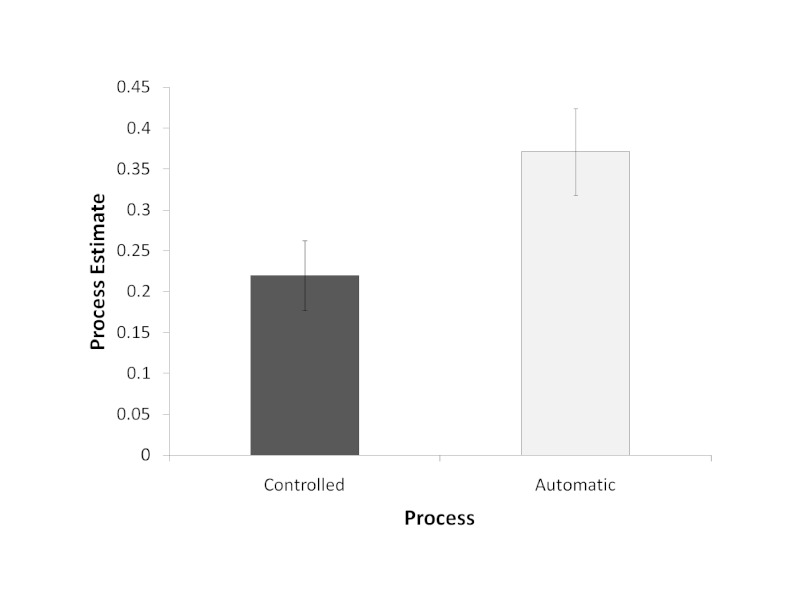
Controlled and automatic process estimates for the recognition test. Error bars represent one standard error.

As per the PDP, the controlled and automatic processing estimates were calculated using the formulas presented in the Introduction for the to-be-excluded sequence. These rates can only be calculated for the to-be-excluded sequence because this is the only sequence in which there can be failures of controlled processes allowing the trained automatic processes to influence responses. The process estimates were submitted to separate *t*-tests to examine whether they were greater than zero. Both the controlled processing estimate (.22), *t*(45) = 5.12, *p* < .05, Cohen’s d = 1.09, and automatic processing estimate (.37), *t*(45) = 7.02, *p* < .05, Cohen’s *d* = 1.50, were greater than zero, as shown in Figure 2.

Participants were separated into those who had greater than zero controlled processing estimates (*n* = 30 of 46, mean controlled processing estimate = .31), and those who did not (*n* = 16, mean controlled processing estimate = 0). This is similar to separating participants on the basis of self-reported awareness of what had been repeating. If learning of the sequence can be supported only by automatic processes, then the participants who do not demonstrate significant controlled processes at retrieval should nonetheless have a significant cost in switching from the trained sequences. The 16 participants who demonstrated no significant evidence of controlled processing showed evidence of learning the sequences. They had a significant cost of transferring to a novel sequence (410 ms) from the trained sequence (388 ms), *t*(15) = 4.33, *p* < .05, Cohen’s *d* = 1.58, consistent with the notion that the sequence could be performed without using controlled processes at test. Training reaction times for participants who failed to demonstrate controlled processing at test, as well as participants who used controlled processing, are shown in Table 3. A second conclusion from this experiment is that having control over the sequence knowledge gained from training is not necessary to receive the reaction time benefit.

**Table 3.** Training Reaction Times for Self-Reported Aware and Non-Aware Participants, as well as Participants Who Did or Did Not Demonstrate Use of Controlled Processing on the Process-Dissociation Test.

		Reaction time	
		Trained	Transfer
Self-report	Non-aware	401 (48)	412 (48)
	Aware	394 (66)	427 (49)
Test processing	Only A	388 (46)	410 (46)
	C and A	401 (67)	429 (49)

*Note*. All reaction times are in milliseconds, and standard deviations are given in parentheses.

To test for a difference between self-report aware and non-aware participants in their ability to control the acquired knowledge, the controlled process estimates were compared between self-reported aware and non-aware participants in a *t*-test. There was not a significant difference in controlled process estimates for the non-aware (*M* = .18)and aware (*M* = .22) participants, *t*(44) = 0.63, *p* > .05, Cohen’s d = 0.21. This suggests that general feelings of awareness of having learned something does not accurately reflect having greater overall controlled processing. This could also be due to an underestimation of the “true” controlled processing occurring in participants, but this would need to be verified by tests with greater sensitivity to processing.

Correlations were run between the transfer cost during training, the self-report measure of awareness, and controlled and automatic process estimates to test for further relations between the different factors. As shown in Table 4, the only significant correlation was between the training transfer cost and self-reported awareness. This small correlation suggests that participants that reported awareness tended to have greater transfer costs. To probe this issue in a more meaningful way, future experiments should use self-report measures that allow for a greater continuum of awareness states. There were no significant correlations between the self-report measure of awareness and the process estimates.

**Table 4.** Correlations Between the Transfer Cost During Training, Self-Reported Awareness, and Process Estimates.

		Self-reported	Process estimate	
	Transfer cost	Awareness	Automatic	Controlled
Transfer cost	—			
Self-report	.37*	—		
Automatic	-.01	-.07	—	
Controlled	.06	.05	-.18	—

*p = .05.

## Discussion

The current study replicated the well-established finding that participants learn a repeated sequence of items within the SRTT paradigm, with shorter latencies in the trained sequences than in the novel sequences encountered towards the end of each training segment. The process estimate data provide evidence consistent with automatic processes operating. However, while the results support a role for automatic processing, the recognition test also shows an influence of controlled processing that highlights the presence of both types of processes in this task.

Implementing the PDP in sequence learning has allowed for a new and different way of examining the behavioral consequences of training participants on repeating sequences. The ability to get through 1,600 trials in 1 hr makes it possible to have participants make a temporal comparison of when they had encountered the probed sequences at test, thus allowing the conditions necessary to meet the PDP assumptions. By having two trained sequences in addition to the new and random test sequences, participants could make exclusion errors thought to be driven by automatic processes (Jacoby, 1991, 1998). Importantly, the trained sequences were not so highly learned or sufficiently distinctive that participants showed perfect recognition. In situations in which participants would be able to recognize all the learned elements as “old”, an absence of necessary errors would then render the PDP inappropriate to use.

The current experiment featured the presence of two learned sequences that needed to be discriminated from each other. Importantly in the context of sequence learning, this rules out the possibility that participants can use perceptual and motor fluency during the recognition test to influence their classification of sequences (see Perruchet & Amorim, 1992). While more fluid execution of a sequence might provide opportunities to distinguish an old sequence from a new one, regardless of whether controlled processes were operating, it would not provide a basis to determine when within the experiment the sequence had been practiced. Previous sequence learning research using the PDP has sought ways to circumvent this issue, for example, through the use of generation of sequences rather than responses to them (Destrebecqz & Cleeremans, 2001), or the addition of further measures (Buchner et al., 1997). However, the design employed here requires minimal variation from the originally developed SRTT method, and hence offers some advantages over previous instantiations of the PDP within sequence learning. For instance, the inclusion of the temporal discrimination at test allows for responses to be made on more than just motor fluency as greater knowledge of when the sequence had been encountered is required.

### The role of awareness

One advantage in investigating implicit learning from an information processing approach is that while knowledge is hard to satisfactorily and exhaustively measure, the underlying processes are more readily testable. In addition to having measures that are more objective than self-report tests, shifting to a processing view of implicit learning also allows for more direct explanations of how the performance changes during and after training. Instead of inferring how the implicit and explicit knowledge types are thought to influence performance, the PDP assesses automatic and controlled processes that support performance. The conditions for how these processes develop and when they tend to be employed can be further investigated using this technique.

Some theoretical accounts of sequence learning (e.g., Keele et al., 2003) already somewhat marginalize the issue of awareness. Awareness may not be a necessary characteristic of any of the processes or systems involved in sequence learning (see also Clegg et al., 1998). We believe conceptualizing performance within implicit learning tasks in terms of the underlying processes, and in particular automatic processing as identified through the PDP approach, provides a means to move beyond debates about the awareness or implicit versus explicit know-ledge. Moreover, the mere ability to impose controlled processing within a task need not indicate that the relevant knowledge for task performance is “explicit”. Examples from motor skill performance show that automatic processes can even be disrupted, and performance degraded, if superseded by conscious monitoring (e.g., Beilock & Carr, 2001).

Further, the fact that participants who lacked controlled processes at test still had a reaction time benefit during training indicates that major use of controlled processes over what has been learned is not necessary. This is in line with definitions of implicit learning that specify the learning can occur without awareness or intent (e.g., Frensch, 1998; Seger, 1994). However, it is possible that even the PDP used in this experiment was insensitive to what controlled knowledge those participants had (Shanks & St. John, 1994). But given the nature and assumptions of PDP (Jacoby, 1991, 1998; Yonelinas & Jacoby, 1994), this seems unlikely. This also again illustrates the utility of moving to a processing account of what changes after training on an implicit learning task. By focusing on the measurable differences in processing, we are no longer reliant on the introspective feelings of awareness as a primary index of how participants are performing the task.

### Future directions

One question for future research is whether recognition tests of the type employed here, and generation tests used in other sequence learning studies (e.g., Destrebecqz & Cleeremans, 2001), are tapping the same underlying processes. In one sense this issue can be related to Shanks and St. Johns (1994) information criterion: whether information used within the test is tapping the information involved during the execution of the actual task. As shown with perceptual priming (e.g., Rajaram & Roediger, 1993), comparing different tests can help inform theory of the underlying processes or testing strategies by looking for similarities and differences in the tests leading to differences in performance. It may also offer insights into any strategic differences between participants on different forms of tests.

Further, while it may seem that previous experiments using generation and recognition tests for SRTT (e.g., Shanks & Johnstone, 1999) have contradictory findings, closer examination reveals that the results are in agreement. Shanks and Johnstone trained participants on similar sequences to the ones used in the current experiment, then administered a free-generation or recognition test. Their results for both re-cognition and free-generation test methods indicated that participants had some explicit knowledge of what had been learned. In the current experiment, we probed the broad category of explicit knowledge further by using the PDP to tease apart the processes supporting the apparent explicit knowledge at test. We chose to implement only a re-cognition test as it guaranteed that participants were being re-exposed to the trained sequences during test, thus forcing them to discriminate between them in their decisions.

Another issue for future research concerns the question whether the temporal discrimination used in this experiment led to an underestimation of the controlled processing estimate since there may exist control over the sequence knowledge itself independent of which half the sequence was occurred in. It is possible that this may be an infrequent occurrence (as concluded by Yonelinas & Jacoby, 1994), or could warrant further methods to allow for valid PDP comparisons.

The results of the correlations between transfer cost, self-reported awareness, and process estimates also suggest further experiments probing these relationships. It is possible that the two-alternative measure for self-reported awareness helped to inflate the correlation with transfer cost. Future experiments should employ a self-reported awareness measure with more responses to test if the correlation still holds with transfer costs.

### Conclusions

Leveraging insights from implicit memory research can provide a foundation for progress on implicit learning. The PDP approach may help implicit learning research move away from arguing over the semantics of what is meant by implicit or explicit and back into the interesting nature of learning processes by providing a way to measure the likely underlying processes. This experiment demonstrated one possible method of using the process estimates to measure the relative contributions of automatic and controlled processes. Additionally, the process estimates were then used to then try to account for differences in training performance. Future improvements, such as non-temporal discriminations, are still possible with the reported technique. SRTT seems to rely on both automatic and controlled processing.

## Footnotes

1 It is possible that participants will lack sufficient control to reliably discriminate the temporal half (Shanks & St. John, 1994). However, even if this is the case, since PDP allows for the calculation of relative use of the two processes, the process estimates will reflect the use of controlled processing if participants are able to discriminate temporal half at least some of the time.

2 This admittedly comes from a field other than sequence learning. However, they argued that their results should generalize to other forms of memory.

3 It is possible that the recognition PDP test will underestimate the controlled process estimate. However, the process estimates are relative estimates of the two process types, so if the controlled process estimate is underestimated the automatic process estimate will be overestimated. But because the two process estimates are mathematically linked, it is incorrect to directly compare the two process estimates. To spoil the results, a controlled process estimate was found, so the concern that a false rejection of controlled processes occurring is unwarranted.

4 These proportions should not be confused with accuracy. These are the proportion of how often each item type was identified as “old”. The concept of a base error rate derived from accuracy is not as important for PDP as the participants’ base rate of incorrectly identifying novel information as having been encountered before. If the false alarm rates differed between the inclusion and exclusion tests for these novel items, it would indicate that participants are using different strategies between tests. If this were true, it would be inappropriate to calculate process estimates.

## Author Note

The authors wish to thank the reviewers for their helpful comments in producing this document. This research is from a thesis submitted to the Academic Faculty of Colorado State University in partial fulfillment of the requirements for the degree of doctor of philosophy.
